# Developing the University of Tartu in Estonia into a well-networked Patient Safety Research Centre (PATSAFE): A study protocol

**DOI:** 10.12688/openreseurope.15024.1

**Published:** 2022-09-05

**Authors:** Kaja PÕLLUSTE, Hiske Calsbeek, Carola Orrego, Marta Ballester, Rosa Suñol, Helena Vall-Roqué, Mari Kangasniemi, Siim Läänelaid, Joel Starkopf, Anne van Tuijl, Hub Wollersheim, Tiina Freimann, Liisi Mägi, Joaquim Bañeres, María del Mar Fernández-Maillo, Yvette Emond, Margus Lember

**Affiliations:** 1 Institute of Clinical Medicine, Faculty of Medicine, University of Tartu, Tartu, 50406, Estonia; 2 IQ healthcare, Radboud Institute for Health Sciences, Radboud university medical center, Nijmegen, 6500 HB, The Netherlands; 3 Avedis Donabedian Research Institute (FAD), Barcelona, 08037, Spain; 4 Universitat Autònoma de Barcelona, Barcelona, 08037, Spain; 5 Health Services Research Network on Chronic Diseases (REDISSEC), Barcelona, Spain; 6 Network for Research on Chronicity, Primary Care, and Health Promotion (RICAPPS), Barcelona, Spain; 7 Institute of Family Medicine and Public Health, Faculty of Medicine, University of Tartu, Tartu, 50411, Estonia; 8 University of Turku, Turku, 20500, Finland; 9 Tartu University Hospital, Tartu, 50406, Estonia; 10 Tartu Health Care College, Tartu, 50411, Estonia

**Keywords:** Patient safety, research competences, early-stage researchers, research strategy, knowledge exchange.

## Abstract

**Background:** Patient safety (PS) is a serious global public health problem affecting all countries. Estimates show that around 10 percent of the patients are harmed during hospital care, resulting in 23 million disability-adjusted life years lost per year. Experts emphasize research advancements as a key precondition for safer care.

**Aim:** The Patient Safety Research Centre (PATSAFE) project enhances the Institute of Clinical Medicine of the University of Tartu’s (ICM-UT) research potential and capacities in PS in order to improve and strengthen knowledge and skills in methods, techniques and experience for PS research.

**Methods:** A strategic partnership with Avedis Donabedian Research Institute in Spain, and IQ Healthcare in the Netherlands, both international leaders in PS research, enables the development of a long-lasting knowledge exchange, allowing the ICM-UT to capitalise on its current achievements and to overcome gaps in scientific excellence in the field of PS research. These twining activities will strengthen and raise the research profile of the ICM-UT academic staff and early-stage researchers (ESRs), by implementing the hands-on training on methods, techniques, and experience in PS research. The project also encourages the active participation of ESRs in PS research by increasing their soft skills, to ensure the continuity and sustainability of PS research in ICM-UT. Finally, development of the research strategy on PS contributes to the long-term sustainability of PS research in Estonia. To implement these activities, PATSAFE foresees a comprehensive strategy consisting of knowledge exchange, soft research skills capacity building, strategic planning, and strong dissemination and exploitation efforts.

**Expected results:** As a result of the project, ICM-UT will have the capacity to carry out PS research using the appropriate methodology and the competences to apply state-of-the-art evidence-based strategies for PS research.

## Introduction

Patient safety (PS) represents a serious global public health problem which affects all countries worldwide. Estimates show that there are 421 million hospitalisations in the world annually, and approximately 42.7 million adverse events, i.e., one in 10 patients is harmed while receiving hospital care. These adverse events result in 23 million disability-adjusted life years lost per year, thus, adverse events due to clinical care could be considered a relevant source of morbidity and mortality globally ^1^.

The publication of ‘To Err is Human’ ^2^ by the American Institute of Medicine in 2000 helped launch the field of PS: an issue of growing professional awareness was converted to one of public concern. Shortly after this report, PS research became an international priority ^3^.

Research is an essential cornerstone for tackling the alarming situation in PS, and a key precondition for safer care. As well as helping to understand the magnitude and nature of patient harm and focus on critical improvement areas, it also contributes to devising evidence-based strategies and evaluating the effectiveness of potential solutions. Thus, using different research methods and approaches, research initiatives in PS focus on three different stages: identification of risks and hazards; design, implementation, and evaluation of PS practices; and maintaining a safe environment and PS culture ^4^.

In 2008, WHO Patient Safety published the global priorities for PS research ^5^, followed by a set of core competencies for PS research ^6^, and a guide for developing training programmes in PS research ^7^. To complement the WHO’s PS initiatives, in June 2009 the Council of the European Union (EU) published recommendations on PS ^8^, calling on Member States to support the establishment and development of national policies and programmes on PS, and develop and promote research on PS. Moreover, the most recent definition of patient safety refers to the importance of an evidence-based approach: “Patient safety is a framework of organized activities that creates cultures, processes, procedures, behaviours, technologies, and environments in health care that consistently and sustainably: lower risks, reduce the occurrence of avoidable harm, make error less likely and reduce its impact when it does occur.” ^9^

Provision of safe and high-quality health services was one of the priorities in the National Health Plan of Estonia 2009-2020 ^10^, and the importance of PS in the Estonian health system is also significantly emphasized in the National Health Plan 2021-2030 ^11^. Quality efforts started in Estonia in the second half of the 1990s with a focus on patients and professionals’ satisfaction. A motive for the further development was the Quality Policy for Estonian Health Care, which was published in 1998 ^12^. In 2002, a set of legislative acts came into force supporting the further development of healthcare quality – basic requirements for the quality and accessibility of health services, minimum standards for health care staff, equipment and rooms to establish the quality of the structure, and some procedural requirements. To adjust to these requirements, most health organizations have introduced and continually developed quality management systems. In general, a lot of attention has been paid to organizational management, occupational safety, and risk management in working environments, and to the patient-centred approach: assessment and documentation of patient health risks, and the implementation of patient satisfaction surveys and complaint management ^13, 14^. However, as suggested by World Bank experts in 2015 ^15^ “a much more fundamental change may be needed in Estonia to create a culture that is open to acknowledging errors and failures, and willing to make the necessary modifications in practice to achieve quality improvement.” Technological improvements alone, without this fundamental behavioural change, will do very little to assure and improve quality. This kind of change could be achieved if healthcare professionals are well trained in the principles of performance measurement, quality improvement, and especially in patient safety and risk management that are specific to their practice specialties. Research on patient safety and implementation of evidence-based approaches will be key to enhance clinicians’ interest and involvement and can also make a difference in practical health care.

Even though the Estonian PS strategy is still not formulated at the national level, a number of initiatives dealing with PS have already been launched, e.g., incident reporting systems in hospitals and pilot record reviews, ^16^. Still, there are no common standards for incident reporting in Estonia, and the collected information is hardly methodically analysed and used for safety improvement. Successful implementation of planned initiatives will require evidence-based information about PS events to assess the existing situation, identify risks to patients, and find the most effective solutions to improve PS and safety culture in general. Therefore, development of research capacity is envisaged as the key driver for enhancing Estonia’s capacity to develop PS.

The Faculty of Medicine of the University of Tartu is Estonia’s only medical school. Within the Faculty, the Institute of Clinical Medicine (ICM-UT) is responsible for most of the clinical subjects of the Medicine programmes (except family medicine and dentistry) and has a leading role in clinical research. Currently, research on health care quality and safety covers a variety of topics, including nosocomial infections, antibiotic usage and resistance, complications in surgery and anaesthesiology, and health outcomes. Moreover, the ICM-UT has experience in the research of quality of care from patient perspectives as well as on the system and provider levels. PS research is currently in an early stage, but due to its position in Estonian health care, the ICM-UT is expected to have a leading role in this research area. ICM-UT offers excellent opportunities for research and innovation in PS because of its unique position in Estonia, although taking advantage of these opportunities is currently hindered by gaps in scientific excellence in the fields of specific methods and techniques for PS research.

Before our project design stage, a thorough SWOT analysis was conducted to identify the gaps in the scientific excellence in PS research methodology at ICM-UT. The SWOT analysis ( Table 1) demonstrated that ICM-UT has high scientific excellence in clinical research methodology and good collaboration with stakeholders (providers of health services, the health ministry, and national health insurance), but that its knowledge in PS research methodology should be improved. Moreover, the intensity of networking in PS between the researchers and providers of health services, as well as motivation among practicing clinicians and health care managers to support PS research needed to be strengthened.

**Table 1.** SWOT analysis – identification of gaps in scientific excellence at Institute of Clinical Medicine of the University of Tartu (ICM-UT).

Strengths	Weaknesses
1. Unique position of ICM-UT in the Estonian medical education and research system. 2. Multidisciplinary early-stage and experienced research staff with excellent clinical competence. 3. Scientific excellence in clinical research methodology, internationally known and experienced researchers. 4. Excellent new infrastructure. 5. Positive attitude from the leading staff of the faculty and institute. 6. Close collaboration with the two leading hospitals – Tartu University Hospital and North Estonia Medical Centre as well with professional associations. 7. Innovative eHealth environment of Estonia.	1. Limited knowledge and skills in patient safety research methodology. 2. Lack of international visibility in the field of patient safety research. 3. Limited number of medical doctors and nurses who have enough knowledge and skills in patient safety research. 4. Scepticism towards the patient safety research and data collection about the risks and hazards of patient safety among practicing clinicians. 5. Low involvement of patients in health care safety research.
Opportunities	Threats
1. Well-trained early-stage researchers willing to contribute to patient safety research. 2. Variety of knowledge and expertise existing in the EU and globally. 3. Close collaboration with and support from the Ministry of Social Affairs and National Health Insurance Fund. 4. Alignment with EC recommendation (2009/C 151/01). 5. High quality health services and patient safety are priorities of the national health strategy. 6. Needs of society (pressure from patients). 7. Unique possibility to link the national eHealth system and data about the risks and hazards of patient safety.	1. Insufficient funding to further develop existing research competence and strengthen the collaboration between scientists and the medical community. 2. Insufficient/unfavourable legislative framework for the implementation of patient safety research in practice. 3. Poor safety culture and resistance from practicing physicians and nurses concerning risk and hazard data collection.

In this context, the Patient Safety Research Centre (PATSAFE) project was designed to improve and strengthen the ICM-UT’s research excellence in the field of PS research among early-stage researchers (ESRs) and academic staff, with a special focus on the improvement of knowledge and skills in methods, techniques, and experience for PS research. The project will guide and improve ICM-UT’s research efforts, support closer cooperation with leading European institutions, and foster the participation of Estonian researchers, health care professionals and policy advisors in international research cooperation and development.

To achieve the project’s main goal, more detailed specific objectives were defined:

1. To strengthen the scientific and technological capacity of ICM-UT and raise ESRs and staff research profile in the field of identification and measurement of risks and hazards in PS.
2. To increase research capacity and to ensure the continuity and sustainability in PS research at ICM-UT by focusing on patient safety culture and patient empowerment regarding their safety.
3. To increase the soft skills of ESRs in order to provide additional competences and improve existing skills in research methods, as well as in research management, including proposal writing, research ethics, research results, intellectual property rights and commercialization, and clinical human resources management.
4. To increase the visibility of ICM-UT’s excellence in PS research and its potential as a partner with internationally leading European and global research and policy counterparts, as well to strengthen ICM-UT´s networking capacity and credibility at the national and international levels.

## Methods

### Ethics policies

The PATSAFE project activities do not raise any ethical issues. As the H2020 WIDESPREAD Twinning call does not cover research and associated costs (which may require various ethics permits) as eligible, such costs and activities have not been applied to the PATSAFE project. On the contrary, by training activities supported by the PATSAFE project, we are raising awareness among project target groups (students on all levels, and research, clinical and teaching staff) on how to conduct ethical research and provide for PS. In case partners identify the need for an ethics permit at any time during the PATSAFE implementation, the permit will be applied for and implementing related actions will be postponed until the permit is granted.

### Project concept

**Figure 1.**
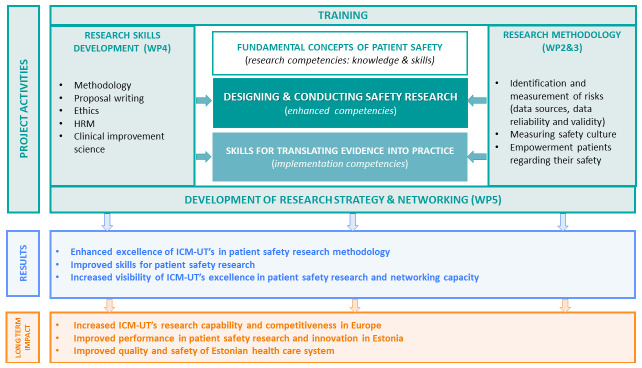
Conception of the Patient Safety Research Centre (PATSAFE) project.

The concept of this project is based on the core competencies for PS research ^6^ as well as on the guide for developing training programmes in PS research ^7^ defined by the WHO’s PS branch. The core competencies for PS research – fundamental concepts of PS, designing and conducting PS research, and putting research evidence into practice – are proposed as a foundation for strengthening research capacity by guiding the development of training programmes for researchers in PS. PS researchers should be able to describe the fundamental concepts of the science of PS in their specific social, cultural, and economic context, design and conduct PS research, and be part of the process of translating research evidence to improve the safe care of patients. The basic concepts of patient safety are discussed during the under- and postgraduate training of health professionals; also, the continuous professional training courses focused on patient safety are available for health staff. Thus, this project does not focus on this part of core competencies and mainly focuses on the development of competencies in methods, techniques, and experience for PS research among the ICM-UT’s ESRs and staff. To ensure the sustainability and continuity of PS research in the future, competencies required for the successful translation of evidence into practice, and crucial research aspects (e.g., intellectual property rights of research results, human resources, and change management in clinical settings) will be included in the training program. These objectives will be achieved through training and research strategy development, the results of which will be expressed as an increase in scientific excellence in PS research and the increased visibility of ICM-UT. The increased level of soft skills will promote the participation of ESRs in further PS research and thereby contribute to the achievement of the long-term impact of the PATSAFE project ( Figure 1).

Considering the SWOT analysis results, the PATSAFE twinning partnership activities involve continuous, peer-to-peer collaboration, training academic staff and ESRs, and networking and coordination activities. Looking ahead, active involvement of ESRs in the project, development of the national research strategy on PS, and establishment of the Estonian Patient Safety Research Network will ensure the long-term sustainability of PS research in ICM-UT and Estonia as a whole.

### Overall methodology

**Figure 2.**
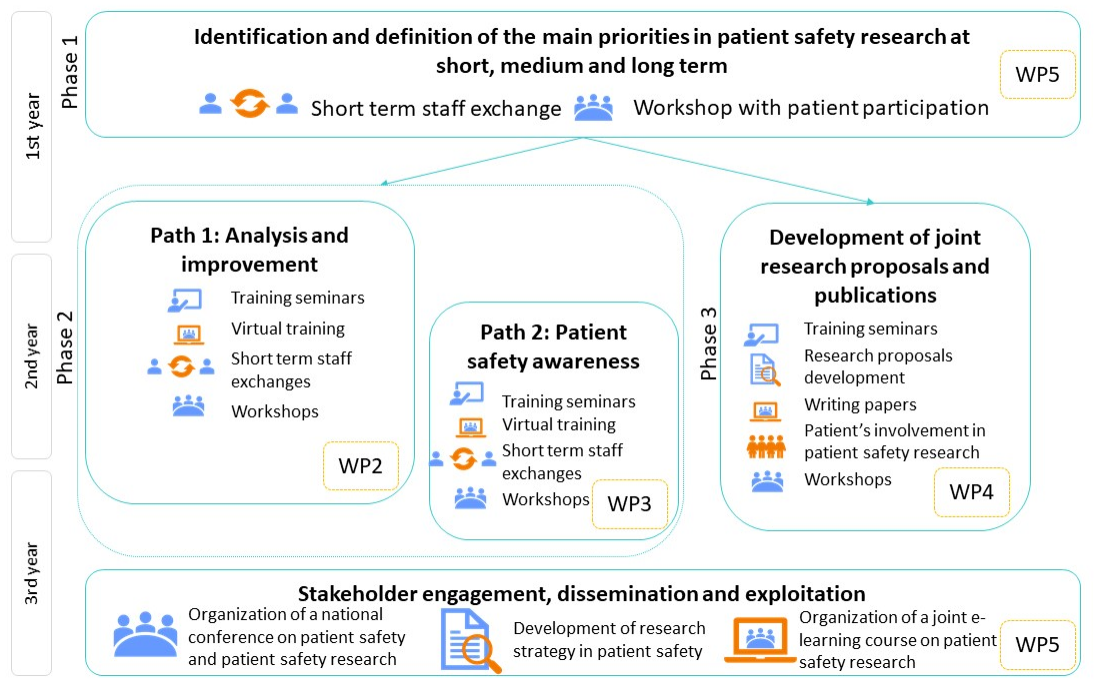
The overall methodology of the Patient Safety Research Centre (PATSAFE) project.

The PATSAFE twinning partnership model is based on continuous, peer-to-peer collaboration, training, networking, and coordination activities. PATSAFE is based on a dynamic and interactive process with iterative improvement cycles and feedback, ongoing communication, incorporation of new ideas and insight and peer-to-peer exchange and focuses on increasing capacities and achieving impact goals ( Figure 2).

**Figure 3.**
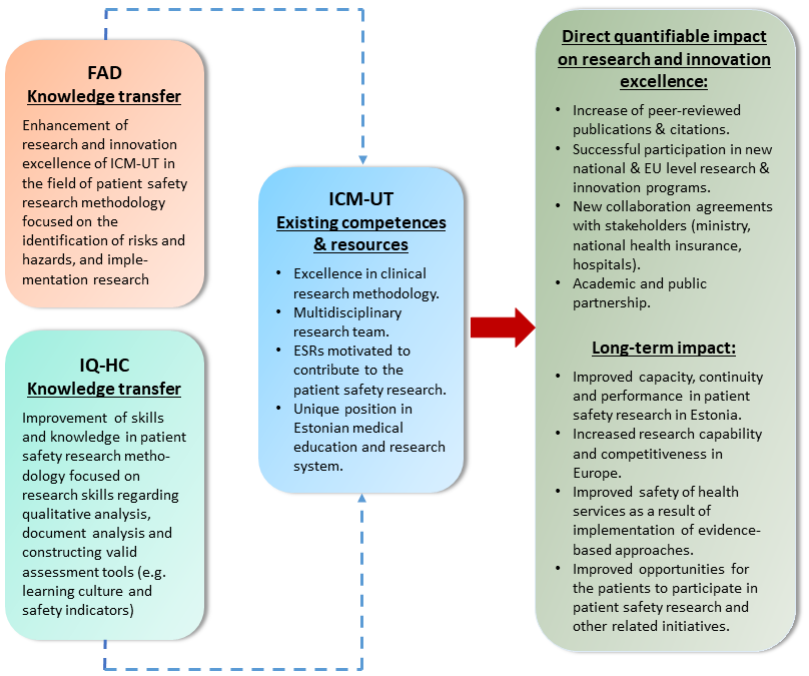
Improvement of research excellence of ICM-UT in patient safety research methodology.

Advanced cooperation between two international leading research centres – the Avedis Donabedian Foundation at the UAB in Barcelona (FAD), and IQ healthcare at the Radboud Institute for Health Sciences, Radboud University Medical Centre (IQ-HC) – and the research organization of the wider region – will help ICM-UT address gaps in PS research *via* knowledge and experience transfer, ( Figure 3), from 2019 to 2022. Identifying common research interests and exploring synergies and knowhow to address specific research questions will pave the way for achieving the ICM-UT’s sustainable development.

### Project activities

The activities of the project are planned according to the findings of the SWOT analysis, aiming to deliver maximum impact on ICM-UT's scientific excellence and to contribute to ICM-UT’s long-term research sustainability.

***1. Identification and definition of the main priorities in short-, medium-, and long-term PS research***

These activities are planned to increase the research capacity and visibility of the ICM-UT in PS research, and directly address the lack of international visibility in the field of PS and low involvement of patients in health care safety research. In the short term, staff exchanges of two-three researchers from the ICM-UT will visit FAD and IQ-healthcare to identify and envisage research topics that are currently addressed at these institutions and at the international level, and how they are implemented. We identify the researchers who have interest in doing PS research or are already involved in PS research. The identification of research topics is based on the national and international priorities in PS research: both the experts on national as well international level will be involved – representatives of researchers, practitioners, patients and policy-makers. To prioritize the research topics, we will use the Delphi technique. Additionally, a multidisciplinary workshop will be organized by the end of the second project year to reach a consensus in prioritization of research topics, including qualitative techniques and a prioritization matrix to find the main short-, medium-, and long-term research needs and challenges. The results will include a list of specific topics, levels of care, and methodologies that are most important, appropriated and adapted to the ICM-UT and Estonia. In this process, patients are involved, to integrate their perspective into the priorities definition of research topics that mostly focus on the procedures and outcomes most important for them. We will involve the patients through their representative organizations by inviting the patient representatives to take part in the priority setting for patient safety research. We will contact the potential participants using the publicly available contact information; the participation is based on the voluntary basis for all participants, including the patients’ representatives. Therefore, the ethical approval is not needed.

***2. Implementing the training program in PS research methodology***

The PATSAFE training program structure features two pathways. Path one focuses on methods for analysis and improvement, such as measuring and analysing risks and hazards, PS improvement interventions, and implementation research. Path two involves methods for researching PS awareness: patient safety culture and patient safety empowerment. This research methodology training program integrates the ‘learning by doing’ approach, using traditional training lectures, with more interactive and participatory activities, combined with mentoring and peer-to peer exchange, in order to work in parallel with the development of specific scientific outputs (proposal, projects, publications, etc.).

Implementing the training program includes face-to-face training seminars, virtual training, short-term staff exchanges, and workshops.

The development of the training program is based on a constructivist view on learning; the principles from adult learning and the flipped classroom approach are used to develop the learning materials and activities.

The learning views from constructivist learning, adult learning and flipping the classroom are translated into the following concrete principles of the program.

- All courses consist of a basic and an advanced version. Participants can choose which version they want to follow based on their learning goals or prior experienced, supporting their self-directed learning. workshops of the course they want to follow based on their learning goals or prior experiences, supporting their self-directed learning.
- Including activities and assignments that have a close link to participants’ workplace and interests.
- Performing activities and assignments in collaboration with colleagues or peers.
- Providing feedback on the assignments from both PATSAFE teachers as from peer participants (peer feedback).

The training program is developed in several steps. First, a needs assessment is undertaken to get insight in the learning needs and preferences regarding the content and learning activities of potential participants. Based on this assessment, general learning goals regarding the main themes (fundamental of patient safety, safety culture & patient involvement, soft skills) are formulated by the curriculum development team. This team compromises of experts on various patient safety topics and healthcare quality topics, researchers, a research ethicist, an educational advisor and technical administrators

Based on the general learning goals, the program is structured in three modules (fundamentals of PS, safety culture & patient involvement and soft skills development) and each module consists over several courses. The courses in the program have different educational formats based on the overall learning goal: full online courses provided on an online learning platform with no live interaction with teachers and courses that include multiple workshops in small groups. Multiple learning methods are used such as reading literature, watching webinars from experts, performing practical and research assignments, doing knowledge tests and being active on forums. All teachers in courses are experts related to a topic on PS.

For every course a flyer is developed and spread amongst potential participants: academic staff, ESRs, health care practitioners. These flyers include a link on which participants could enroll to the course and get access to the learning materials. The flyers are spread in the different mailing lists in the Faculty of Medicine of the University of Tartu and Faculty’s information letter, on the project webpage and university’s continuous education program.

Theoretical topics from paths one and two will be addressed by face-to-face interactive seminars, including case studies and discussions. For the virtual training, all the materials prepared are organized *via* modules and pathways using a Moodle e-learning platform. This e-learning platform is also used to promote exchange and facilitate work between face-to-face activities. The described activities focus on strengthening the scientific and technological capacity to identify and measure risks and hazards in PS, PS culture, and empowering patients to improve their own safety. They address the weaknesses that were revealed in the SWOT analysis: limited knowledge in patient safety research methodology among the ICM-UT research staff and ESRs, and a limited number of medical doctors and nurses with enough knowledge and skills in patient safety research. To assess how the knowledge and skills regarding PS research improved during implementation of the program, various assessments are undertaken. Depending on the content and goals of the different learning modules, an assessment method is used such as knowledge tests, practical activities or written assignments.

***3. Development of a joint research proposal and publications***

Poor safety culture might be a serious obstacle for PS research, as the quality of research results depends to a great extent on the readiness of clinicians to support the research process. Thus, research in safety culture indicates obstacles to the willingness of staff to participate in research processes or opportunities to improve safety culture and thus promote patient safety research.

Research activities are guided by the WHO PS research priorities and competencies and will directly address weaknesses like scepticism towards PS research and data collection, and limited knowledge in PS research methodology among the ICM-UT research staff and ESRs.

Using topics prioritized in phase one and implemented in parallel with phase two, face-to-face interactive training seminars will be organized in phase three to address different soft skills needed to ensure that research topics are correctly translated into specific outputs. At least three proposals will be prepared during the life span of the project. The participants of these training seminars are the members of the academic staff as well the ESRs who participate in the training program and are interested to write the research proposal.

Writing the scientific papers will also be a means to integrate training, mentoring, and exchange into specific scientific output. With this, different types of skills are practiced.

During the implementation of this project, ICM-UT staff’s and ESR competency in the qualitative and quantitative research methods for patient involvement and empowerment for PS research are expected to increase. This activity addresses weaknesses like low involvement of patients in health care safety research and limited knowledge in PS research methodology among the ICM-UT research staff and ESRs.

Workshops are organized as follow-up activities to the aforementioned activities.

The research activities address the following weaknesses: lack of international visibility in PS research, scepticism towards PS research and data collection about the risks and hazards of PS among practicing clinicians, and the low involvement of patients in health care safety research. To assess improvement, we define the indicators that are described in detail in the Impact section of this protocol.

***4. Stakeholder engagement, dissemination, and exploitation***

This section coordinates the engagement of relevant stakeholders and implements an ambitious plan of innovative activities to increase the impact of the project research. These activities directly address weaknesses like the lack of international visibility and scepticism towards PS research and data collection.

This phase covers the integration of PS research into the development plans of the ICM-UT, the Faculty of Medicine of the University of Tartu, and the Estonian National Health Plan. Elaborating the long-term strategic development plan, and involving relevant stakeholders, will ensure maximum synergies with national and European priorities, maximize the effects of structural funds, and ensure that research and innovation resources attain critical mass. Moreover, the strategy will increase the visibility of ICM-UT’s scientific excellence and its potential as an equal partner within European academia and health politics. At the end of the project, we will organize a national conference on PS and PS research. As an exploitation strategy, the consortium will take advantage of the material, methodology, and experience developed during phases one, two, and three and organize a virtual training course on PS research open to other potential interested professionals in Europe. To carry out the course implementation, we will perform a business case study, combining marketing, diffusion, and launching.

## Impact

The PATSAFE project will substantially and measurably improve scientific and innovation capabilities and the performance of ICM-UT in PS research methodology. Thus, it will also improve Estonia’s PS research and innovation, and overall health care quality ( Figure 3). The current data suggest that about one in 10 patients is harmed while receiving hospital care, and about 15% of hospital expenditure and activity in Organisation for Economic Co-operation and Development (OECD) countries can be attributed to treating safety failures ^17^. Currently, there are no reliable data about the prevalence of healthcare-related patient harm in Estonia, but adjustment of the international data to Estonian health system demonstrates that in 2016 about 21,675 patients or 1,647 per 100,000 inhabitants could potentially have been harmed during their hospital stays, and that about 98,850,000 Euros were spent treating these failures. These calculations are based on the data available at: https://statistika.tai.ee/pxweb/en/Andmebaas/Andmebaas__04THressursid/

Increasing Estonian PS research capabilities and performance will enable researchers to investigate the magnitude and nature of patient harm in Estonia and, ultimately promote the development of evidence-based strategies and evaluating the effectiveness of potential solutions. This approach will eventually decrease the high burden on healthcare-caused harm to the loss of capacity and productivity of patients being harmed, and to the loss of trust in the health system, and lead to additional available resources within the health system ^18^.

Increased research excellence, improved scientific and innovation capabilities, and better performance by the ICM-UT are expected to be revealed through the following indicators:

- An increase in peer-reviewed publications and citations;
- increased visibility – increased number of submitted, accepted and invited presentations in international events;
- successful collaboration with stakeholders – providers of health services, the Estonian Health Insurance Fund, and the Ministry of Social Affairs;
- successful participation in new national or EU level competitive research and innovation programmes;
- new and improved courses available in person or online.

The expected long-term impact of the PATSAFE project is defined as follows:

- Increased European research capabilities and competitiveness;
- improved capacity and performance in Estonian PS research and innovation; and
- improved quality and safety within the Estonian health care system.

The primary objective of the long-term programme in PS is to create PS knowledge that will improve the safety of health services at the individual and population levels by providing evidence-based strategies. The ICM-UT outputs will:

- Enable the country-wide assessment of PS incidents and risk assessment;
- contribute to the development of clinical and PS risk analysis and risk management;
- increase public awareness in PS;
- reduce the burden of patient harm caused by healthcare.

Thus, ICM-UT research in PS will have a high impact on the overall healthcare quality and safety. The ICM-UT will bring together national and international experts to exchange knowledge and experience, and thereby maximise the impact of the research for the benefit of the patients and clients of health system.

## Conclusion

As a result of the project, the ICM-UT will apply state-of-the-art evidence-based strategies to PS research. The ICM-UT will have the capacity to conduct PS research using appropriate methodology, promote PS research among ESRs and healthcare staff, and involve patients in PS research, thus contributing to Estonia’s overall health care quality and PS performance. For partner institutions, participation in this project provides new opportunities for networking and expanding their research methods to a new culture and setting. 

## Data availability

### Underlying data

No data are associated with this article.
