# Developing the University of Tartu in Estonia into a well-networked Patient Safety Research Centre (PATSAFE): A study protocol

**DOI:** 10.12688/openreseurope.15024.2

**Published:** 2023-11-24

**Authors:** Kaja PÕLLUSTE, Hiske Calsbeek, Carola Orrego, Marta Ballester, Rosa Suñol, Helena Vall-Roqué, Mari Kangasniemi, Siim Läänelaid, Joel Starkopf, Anne van Tuijl, Hub Wollersheim, Tiina Freimann, Liisi Mägi, Joaquim Bañeres, María del Mar Fernández-Maillo, Yvette Emond, Margus Lember

**Affiliations:** 1 Institute of Clinical Medicine, Faculty of Medicine, University of Tartu, Tartu, 50406, Estonia; 2 IQ healthcare, Radboud Institute for Health Sciences, Radboud university medical center, Nijmegen, 6500 HB, The Netherlands; 3 Avedis Donabedian Research Institute (FAD), Barcelona, 08037, Spain; 4 Universitat Autònoma de Barcelona, Barcelona, 08037, Spain; 5 Health Services Research Network on Chronic Diseases (REDISSEC), Barcelona, Spain; 6 Network for Research on Chronicity, Primary Care, and Health Promotion (RICAPPS), Barcelona, Spain; 7 Institute of Family Medicine and Public Health, Faculty of Medicine, University of Tartu, Tartu, 50411, Estonia; 8 University of Turku, Turku, 20500, Finland; 9 Tartu University Hospital, Tartu, 50406, Estonia; 10 Tartu Health Care College, Tartu, 50411, Estonia

**Keywords:** Patient safety, research competences, early-stage researchers, research strategy, knowledge exchange.

## Abstract

**Background:**

Patient safety (PS) is a serious global public health problem affecting all countries. Estimates show that around 10 percent of the patients are harmed during hospital care, resulting in 23 million disability-adjusted life years lost per year. Experts emphasize research advancements as a key precondition for safer care.

**Aim:**

The Patient Safety Research Centre (PATSAFE) project enhances the Institute of Clinical Medicine of the University of Tartu’s (ICM-UT) research potential and capacities in PS in order to improve and strengthen knowledge and skills in methods, techniques and experience for PS research.

**Methods:**

A strategic partnership with Avedis Donabedian Research Institute in Spain, and IQ Healthcare in the Netherlands, both international leaders in PS research, enables the development of a long-lasting knowledge exchange, allowing the ICM-UT to capitalise on its current achievements and to overcome gaps in scientific excellence in the field of PS research. These twining activities will strengthen and raise the research profile of the ICM-UT academic staff and early-stage researchers (ESRs), by implementing the hands-on training on methods, techniques, and experience in PS research. The project also encourages the active participation of ESRs in PS research by increasing their soft skills, to ensure the continuity and sustainability of PS research in ICM-UT. Finally, development of the research strategy on PS contributes to the long-term sustainability of PS research in Estonia. To implement these activities, PATSAFE foresees a comprehensive strategy consisting of knowledge exchange, soft research skills capacity building, strategic planning, and strong dissemination and exploitation efforts.

**Expected results:**

As a result of the project, ICM-UT will have the capacity to carry out PS research using the appropriate methodology and the competences to apply state-of-the-art evidence-based strategies for PS research.

## Introduction

Patient safety (PS) represents a serious global public health problem which affects all countries worldwide. Estimates show that there are 421 million hospitalisations in the world annually, and approximately 42.7 million adverse events, i.e., one in 10 patients is harmed while receiving hospital care. These adverse events result in 23 million disability-adjusted life years lost per year, thus, adverse events due to clinical care could be considered a relevant source of morbidity and mortality globally ^1^.

The publication of ‘To Err is Human’ ^2^ by the American Institute of Medicine in 2000 helped launch the field of PS: an issue of growing professional awareness was converted to one of public concern. Shortly after this report, PS research became an international priority ^3^.

Research is an essential cornerstone for tackling the alarming situation in PS, and a key precondition for safer care. As well as helping to understand the magnitude and nature of patient harm and focus on critical improvement areas, it also contributes to devising evidence-based strategies and evaluating the effectiveness of potential solutions. Thus, using different research methods and approaches, research initiatives in PS focus on three different stages: identification of risks and hazards; design, implementation, and evaluation of PS practices; and maintaining a safe environment and PS culture ^4^.

In 2008, WHO Patient Safety published the global priorities for PS research ^5^, followed by a set of core competencies for PS research ^6^, and a guide for developing training programmes in PS research ^7^. To complement the WHO’s PS initiatives, in June 2009 the Council of the European Union (EU) published recommendations on PS ^8^, calling on Member States to support the establishment and development of national policies and programmes on PS, and develop and promote research on PS. Moreover, the most recent definition of patient safety refers to the importance of an evidence-based approach: “Patient safety is a framework of organized activities that creates cultures, processes, procedures, behaviours, technologies, and environments in health care that consistently and sustainably: lower risks, reduce the occurrence of avoidable harm, make error less likely and reduce its impact when it does occur.” ^9^

Provision of safe and high-quality health services was one of the priorities in the National Health Plan of Estonia 2009–2020 ^10^, and the importance of PS in the Estonian health system is also significantly emphasized in the National Health Plan 2021–2030 ^11^. Quality efforts started in Estonia in the second half of the 1990s with a focus on patients and professionals’ satisfaction. A motive for the further development was the Quality Policy for Estonian Health Care, which was published in 1998 ^12^. In 2002, a set of legislative acts came into force supporting the further development of healthcare quality – basic requirements for the quality and accessibility of health services, minimum standards for health care staff, equipment and rooms to establish the quality of the structure, and some procedural requirements. To adjust to these requirements, most health organizations have introduced and continually developed quality management systems. In general, a lot of attention has been paid to organizational management, occupational safety, and risk management in working environments, and to the patient-centred approach: assessment and documentation of patient health risks, and the implementation of patient satisfaction surveys and complaint management ^13, 14^. However, as suggested by World Bank experts in 2015 ^15^ “a much more fundamental change may be needed in Estonia to create a culture that is open to acknowledging errors and failures, and willing to make the necessary modifications in practice to achieve quality improvement.” Technological improvements alone, without this fundamental behavioural change, will do very little to assure and improve quality. This kind of change could be achieved if healthcare professionals are well trained in the principles of performance measurement, quality improvement, and especially in PS and risk management that are specific to their practice specialties. Research on PS and implementation of evidence-based approaches will be key to enhance clinicians’ interest and involvement and can also make a difference in practical health care.

Even though the Estonian PS strategy is still not formulated at the national level, a number of initiatives dealing with PS have already been launched, e.g., incident reporting systems in hospitals and pilot record reviews ^16^. Still, there are no common standards for incident reporting in Estonia, and the collected information is hardly methodically analysed and used for safety improvement. Successful implementation of planned initiatives will require evidence-based information about PS events to assess the existing situation, identify risks to patients, and find the most effective solutions to improve PS and safety culture in general. Therefore, development of research capacity is envisaged as the key driver for enhancing Estonia’s capacity to develop PS.

The Faculty of Medicine of the University of Tartu is Estonia’s only medical school. Within the Faculty, the Institute of Clinical Medicine (ICM-UT) is responsible for most of the clinical subjects of the Medicine programmes (except family medicine and dentistry) and has a leading role in clinical research. Currently, research on health care quality and safety covers a variety of topics, including nosocomial infections, antibiotic usage and resistance, complications in surgery and anaesthesiology, and health outcomes. Moreover, the ICM-UT has experience in the research of quality of care from patient perspectives as well as on the system and provider levels. PS research is currently in an early stage, but due to its position in Estonian health care, the ICM-UT is expected to have a leading role in this research area. ICM-UT offers excellent opportunities for research and innovation in PS because of its unique position in Estonia, although taking advantage of these opportunities is currently hindered by gaps in scientific excellence in the fields of specific methods and techniques for PS research.

We started the preparatory work for the Patient Safety Research Centre (PATSAFE) project by conducting a thorough SWOT analysis to identify the gaps in the scientific excellence in PS research methodology at ICM-UT. The SWOT analysis ( Table 1) demonstrated that ICM-UT has high scientific excellence in clinical research methodology and good collaboration with stakeholders (providers of health services, the health ministry, and national health insurance), but that its knowledge in PS research methodology should be improved. Moreover, the intensity of networking in PS between the researchers and providers of health services, as well as motivation among practicing clinicians and health care managers to support PS research needed to be strengthened.

**Table 1.** SWOT analysis – identification of gaps in scientific excellence at Institute of Clinical Medicine of the University of Tartu (ICM-UT).

Strengths	Weaknesses
1. Unique position of ICM-UT in the Estonian medical education and research system. 2. Multidisciplinary early-stage and experienced research staff with excellent clinical competence. 3. Scientific excellence in clinical research methodology, internationally known and experienced researchers. 4. Excellent new infrastructure. 5. Positive attitude from the leading staff of the faculty and institute. 6. Close collaboration with the two leading hospitals – Tartu University Hospital and North Estonia Medical Centre as well with professional associations. 7. Innovative eHealth environment of Estonia.	1. Limited knowledge and skills in patient safety research methodology. 2. Lack of international visibility in the field of patient safety research. 3. Limited number of medical doctors and nurses who have enough knowledge and skills in patient safety research. 4. Scepticism towards the patient safety research and data collection about the risks and hazards of patient safety among practicing clinicians. 5. Low involvement of patients in health care safety research.
Opportunities	Threats
1. Well-trained early-stage researchers willing to contribute to patient safety research. 2. Variety of knowledge and expertise existing in the EU and globally. 3. Close collaboration with and support from the Ministry of Social Affairs and National Health Insurance Fund. 4. Alignment with EC recommendation (2009/C 151/01). 5. High quality health services and patient safety are priorities of the national health strategy. 6. Needs of society (pressure from patients). 7. Unique possibility to link the national eHealth system and data about the risks and hazards of patient safety.	1. Insufficient funding to further develop existing research competence and strengthen the collaboration between scientists and the medical community. 2. Insufficient/unfavourable legislative framework for the implementation of patient safety research in practice. 3. Poor safety culture and resistance from practicing physicians and nurses concerning risk and hazard data collection.

Considering the SWOT analysis, we designed the PATSAFE project to improve and strengthen the ICM-UT’s research excellence in the field of PS research among early-stage researchers (ESRs) and academic staff. We focused especially on the improvement of knowledge and skills in methods, techniques, and experience for PS research. The project was planned to guide and improve ICM-UT’s research efforts, support closer cooperation with leading European institutions, and foster the participation of Estonian researchers, healthcare professionals and policy advisors in international research cooperation and development.

To achieve the project’s main goal, the following specific objectives were defined:

1. To strengthen the scientific and technological capacity of ICM-UT and raise ESRs and staff research profiles for identifying and measuring risks and hazards in PS.
2. To increase research capacity and to ensure the continuity and sustainability of PS research at ICM-UT by focusing on patient safety culture and patient empowerment regarding their safety.
3. To increase the soft skills of ESRs. Soft skills are the elementary management, personal, and interpersonal abilities that are vital for an individual to be efficient at workplace or in their personal life ^17^. In this project, soft skills are defined as skills that provide additional competencies and improve existing skills in research methods, as well as in research management, including proposal writing, research ethics, intellectual property rights and commercialization, and clinical human resources management.
4. To increase the visibility of ICM-UT’s excellence in PS research and its potential as a partner with internationally leading European and global research and policy counterparts, as well to strengthen ICM-UT´s networking capacity and credibility at the national and international levels.

## Methods

### Ethics policies

The PATSAFE project activities do not raise any ethical issues. As the H2020 WIDESPREAD Twinning call does not cover research and associated costs (which may require various ethics permits) as eligible, such costs and activities have not been applied to the PATSAFE project. On the contrary, through training activities supported by the PATSAFE project, we are raising awareness among project target groups (students on all levels and research, clinical and teaching staff) on how to conduct ethical research and provide for PS. In case partners identify the need for an ethics permit at any time during the PATSAFE implementation, the permit will be applied for and implementing related actions will be postponed until the permit is granted.

### Project concept

**Figure 1.**
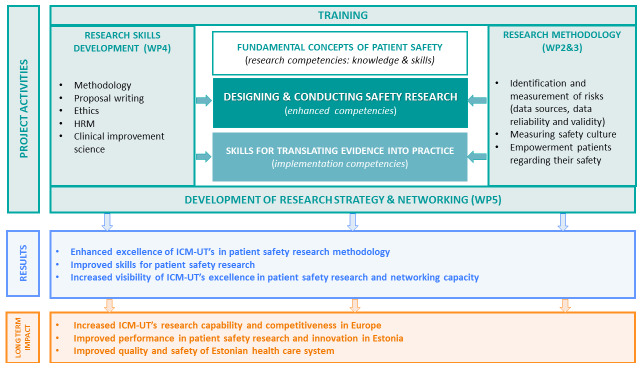
Conception of the Patient Safety Research Centre (PATSAFE) project.

The concept of this project is based on the core competencies for PS research as well as on the guide for developing training programmes in PS research defined by the WHO’s PS branch. The core competencies for PS research, such as fundamental concepts of PS, designing and conducting PS research, and putting research evidence into practice, – are proposed as a foundation for strengthening research capacity by guiding the development of training programmes for researchers in PS. PS researchers should be able to describe the fundamental concepts of the science of PS in their specific social, cultural, and economic context, design and conduct PS research, and be part of the process of translating research evidence to improve the safe care of patients. This project mainly focuses on developing competencies in methods, techniques, and experience for PS research among the ICM-UT’s ESRs and staff. To ensure that the participants in the research training have the same level of knowledge, some basic concepts of PS are introduced as well. To ensure the sustainability and continuity of PS research in the future, the competencies for the successful translation of evidence into practice and crucial research aspects (e.g., intellectual property rights of research results, human resources, and change management in clinical settings) are included in the training program. These objectives are achieved through training and research strategy development. The increased level of soft skills promotes the participation of ESRs in further PS research and thereby contributes to the achievement of the long-term impact of the PATSAFE project ( Figure 1). All project activities are divided into five work packages (WPs).

Considering the SWOT analysis results, the PATSAFE twinning partnership activities involve continuous peer-to-peer collaboration, training academic staff and ESRs, and networking and coordination activities. Looking ahead, the active involvement of ESRs in the project, the development of the national research strategy on PS, and establishment of the Estonian Patient Safety Research Network will ensure the long-term sustainability of PS research in ICM-UT and Estonia as a whole.

### Overall methodology

**Figure 2.**
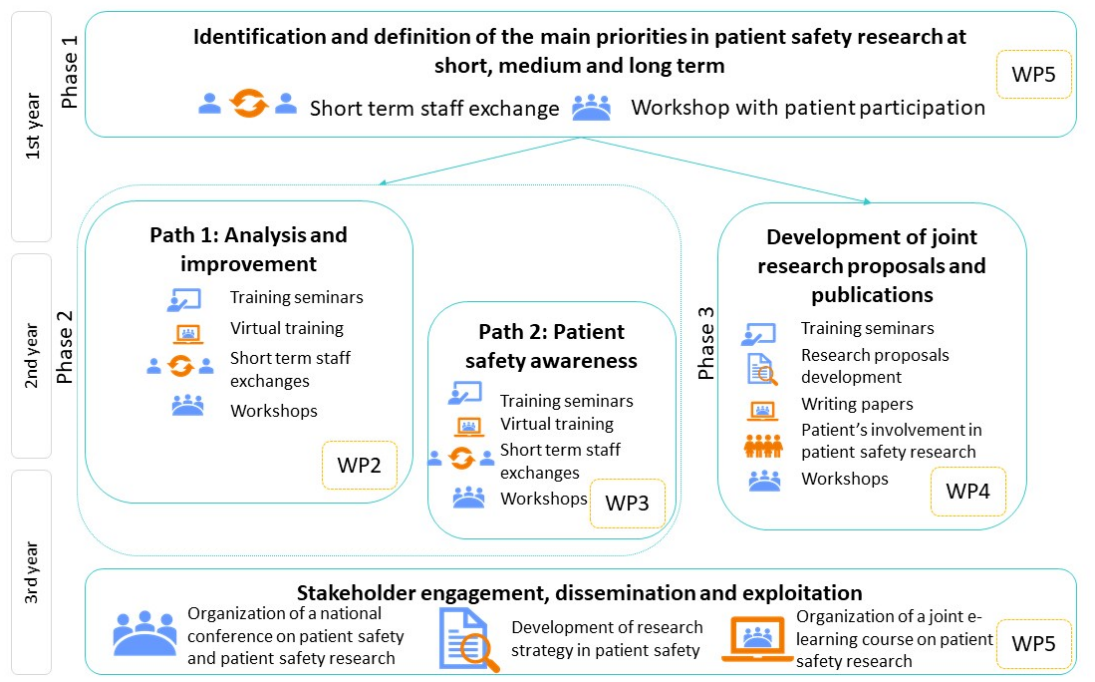
The overall methodology of the Patient Safety Research Centre (PATSAFE) project.

 PATSAFE is based on a dynamic and interactive process with iterative improvement cycles and feedback, ongoing communication, incorporation of new ideas and insight and peer-to-peer exchange and focuses on increasing capacities and achieving impact goals ( Figure 2).

**Figure 3.**
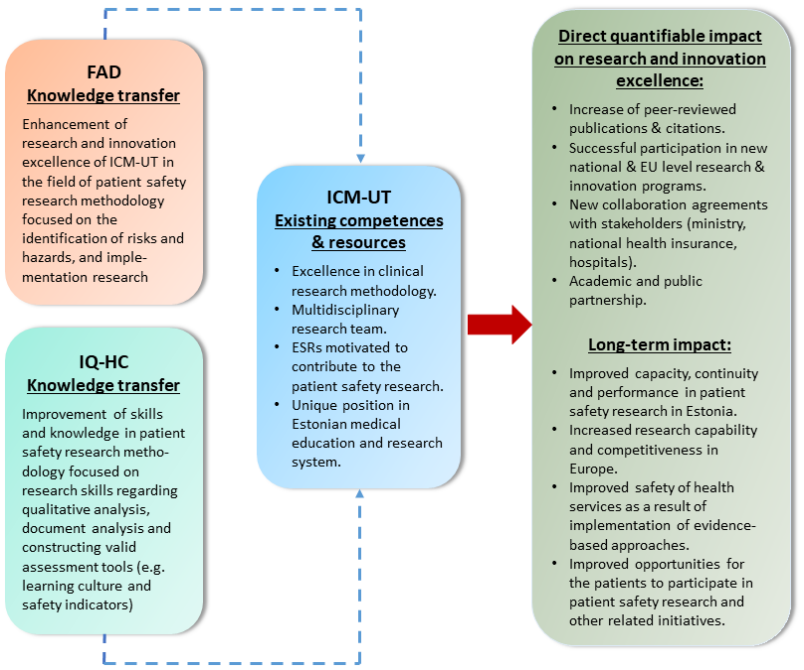
Improvement of research excellence of ICM-UT in patient safety research methodology.

Advanced cooperation between two international leading research centres – the Avedis Donabedian Foundation at the UAB in Barcelona (FAD), and IQ healthcare at the Radboud Institute for Health Sciences, Radboud university medical center (IQ-HC) – and the research organization of the wider region –helps ICM-UT address the gaps in PS research *via* knowledge and experience transfer, ( Figure 3), from 2019 to 2022. Identifying common research interests and exploring synergies and knowhow to address specific research questions paves the way for achieving the ICM-UT’s sustainable development.

### Project activities

PATSAFE project includes four activities, which are planned according to the SWOT analysis findings, to deliver maximum impact on ICM-UT's scientific excellence and contribute to ICM-UT’s long-term research sustainability.

***1. Identification and definition of the main priorities in short-, medium-, and long-term PS research.*** These activities are planned to increase the research capacity and visibility of the ICM-UT in PS research, and directly address the need for international visibility in PS and low involvement of patients in healthcare safety research. In the short term, staff exchanges of two to three researchers from the ICM-UT will visit FAD and IQ-healthcare to identify and envisage research topics that are currently addressed at these institutions and the international level and how they are implemented. We identify the researchers who are interested in doing PS research or are already involved in PS research. Identification of research topics is based on the national and international priorities in PS research, by involving national and international level experts, i.e. representatives of researchers, practitioners, patients and policymakers. To prioritize the research topics, we use the Delphi technique. Additionally, a multidisciplinary workshop is organized to reach a consensus on prioritization of research topics, including qualitative techniques and a prioritization matrix to find the primary short-, medium-, and long-term research needs and challenges. The results include a list of specific topics, care levels, and most important methodologies, appropriated and adapted to the ICM-UT and Estonia. In this process, patients are involved, to integrate their perspective into the priority's definition of research topics that primarily focus on the procedures and outcomes most important for them. We involve the patients through their representative organizations by inviting the patient representatives to take part in the priority setting for patient safety research. We contact the potential participants using the publicly available contact information; the participation is voluntary for all participants, including the patients’ representatives. The prioritization process leads to the development of the national PS research strategy.

***2. Implementation of the training program in PS research methodology.*** The PATSAFE training program structure features two pathways. Path one focuses on methods for analysis and improvement, such as measuring and analysing risks and hazards, PS improvement interventions, and implementation research. Path two involves methods for researching PS awareness: patient safety culture and patient safety empowerment. This research methodology training program integrates the ‘learning by doing’ approach, using traditional training lectures, with more interactive and participatory activities, combined with mentoring and peer-to-peer exchange, to work in parallel with the development of specific scientific outputs (proposal, projects, publications, etc.).

The training program development is based on a constructivist learning theory and principles from adult learning. A flipped classroom approach is used to develop the learning materials and activities. These principles lead to the following structure of the training program:

- All courses consist of a basic and an advanced version. Participants can choose which version to follow based on their learning goals or prior experience, supporting their self-directed learning.
- The program includes activities and assignments closely linked to participants’ workplace and interests.
- Activities and assignments are performed in collaboration with colleagues or peers.
- The feedback on the assignments is provided by PATSAFE teachers and peer participants (peer feedback).

The training program is developed in several steps. First, a needs assessment is undertaken to get insight into potential participants' learning needs and preferences regarding the content and learning activities. Based on this assessment, the transnational curriculum development team formulates general learning goals regarding the main themes. This team comprises experts on various topics concerning PS and healthcare quality, researchers, a research ethicist, an educational advisor and technical administrators from the universities involved in the PATSAFE project.

Based on the general learning goals, the program is structured into three modules, each consisting over several courses:

- fundamentals of PS, including the methods for analysis and improvement, such as measuring and analysing risks and hazards, PS improvement interventions, and implementation research;safety culture and patient involvement, also including validation techniques of measurement tools to study the safety culture in healthcare settings;soft skills development such as writing the research proposal, research ethics, commercialization in research and intellectual property rights, human resources and change management in clinical settings, qualitative research as well the introduction on literature reviews.

The courses in the program have different educational formats based on the overall learning goals. Some are full online courses in an online learning platform with no live interaction with teachers. Others include multiple workshops in small groups. Multiple learning methods are used, such as reading literature, watching webinars from experts, performing practical and research assignments, doing knowledge tests and being active on forums. All teachers in courses are experts related to a topic on PS: academic staff members from FAD, IQ-HC, and ICM-UT but also international leading experts and PS research leaders from other European academic institutions

In order to spread the information about the training program and to recruit the participants, we organize an introductory workshop at the beginning of the project. Additionally, for every course a flyer is developed and spread amongst potential participants: academic staff ESRs, and healthcare practitioners, using mailing lists in the Faculty of Medicine of the University of Tartu and Faculty’s information letter, the project webpage and university’s continuous education program.

Theoretical topics from paths one and two are addressed by face-to-face interactive seminars, including case studies and discussions. For the virtual training, all the materials prepared are organized *via* modules and pathways using a Moodle e-learning platform of the University of Tartu. This e-learning platform is also used to promote exchange and facilitate work between face-to-face activities. The described activities focus on strengthening the scientific and technological capacity to identify and measure risks and hazards in PS, PS culture, and empowering patients to improve their own safety. They address the weaknesses revealed in the SWOT analysis: limited knowledge in PS research methodology among the ICM-UT research staff and ESRs, and a limited number of medical doctors and nurses with enough knowledge and skills in patient safety research. To assess the improvement of knowledge and skills regarding PS research among participants, various assessments are undertaken, such as knowledge tests, practical activities or written assignments.

The training program is implemented over a two-year period starting from 2020 to 2022

***3. Development of a joint research proposal and publications.*** Poor safety culture might be a serious obstacle for PS research, as the quality of research results depends to a great extent on the readiness of clinicians to support the research process. Thus, research in safety culture indicates obstacles to the willingness of staff to participate in research processes or opportunities to improve safety culture and thus promote patient safety research.

Research activities are guided by the WHO PS research priorities and competencies as well by the research priorities defined in Estonia and directly address weaknesses like scepticism towards PS research and data collection, and limited knowledge in PS research methodology among the ICM-UT research staff and ESRs.

Using topics prioritized in phase one and implemented in parallel with phase two, face-to-face interactive training seminars are organized in phase three to address different soft skills needed to ensure that research topics are correctly translated into specific outputs. The participants of these training seminars are the members of the academic staff as well the ESRs who participated in the training program and are interested to write the research proposal. Researchers are encouraged to prepare proposals to fund their own research, but the aim of the consortium is to prepare proposals for the continuation of the collaboration after the end of this project, too. Depending on relevant international or national calls for proposals, up to three proposals are planned during the life span of the project, with one consortium member as lead partner in each proposal.

Writing the scientific papers is also a mean to integrate training, mentoring, and exchange into specific scientific output. With this, different types of skills can be practiced. These activities address weaknesses like low involvement of patients in health care safety research and limited knowledge in PS research methodology among the ICM-UT research staff and ESRs but also weaknesses such as.

lack of international visibility in PS research, scepticism towards PS research and data collection about the risks and hazards of PS among practicing clinicians, and the low involvement of patients in health care safety research. To assess improvement, we define the indicators that are described in detail in the Impact section of this protocol.

***4. Stakeholder engagement, dissemination, and exploitation.*** This activity coordinates the engagement of relevant stakeholders and implements an ambitious plan of innovative activities to increase the impact of the project research. These activities directly address weaknesses like the lack of international visibility and scepticism towards PS research and data collection.

During this activity the integration of PS research into the development plans of the ICM-UT, the Faculty of Medicine of the University of Tartu, and the Estonian National Health Plan is covered. By elaborating the long-term strategic development plan, and involving relevant stakeholders, the effects of structural funds are maximised, and research and innovation resources attainment to critical mass is ensured. Moreover, the strategy will increase the visibility of ICM-UT’s scientific excellence and its potential as an equal partner within European academia and health politics. At the end of the project, we organize a national conference on PS and PS research. As an exploitation strategy, the consortium takes advantage of the material, methodology, and experience developed during phases one, two, and three and organizes a virtual training course on PS research open to other potential interested professionals in Europe. To carry out the course implementation, we perform a business case study, combining marketing, diffusion, and launching.

### Impact

The PATSAFE project substantially and measurably improves scientific and innovation capabilities and the performance of ICM-UT in PS research methodology. Thus, it also improves Estonia’s PS research and innovation, and overall health care quality ( Figure 3). The current data suggest that about one in 10 patients is harmed while receiving hospital care, and about 15% of hospital expenditure and activity in Organisation for Economic Co-operation and Development (OECD) countries can be attributed to treating safety failures ^18^. Currently, there are no reliable data about the prevalence of healthcare-related patient harm in Estonia, but adjustment of the international data to Estonian health system demonstrates that in 2016 about 21,675 patients or 1,647 per 100,000 inhabitants could potentially have been harmed during their hospital stays, and that about 98,850,000 Euros were spent treating these failures. These calculations are based on the data provided by Estonian Health Statistic and HEalth REsearch Database : https://statistika.tai.ee/pxweb/en/Andmebaas/Andmebaas__04THressursid/

Increasing Estonian PS research capabilities and performance enables researchers to investigate the magnitude and nature of patient harm in Estonia and, ultimately, promote the development of evidence-based strategies and evaluate the effectiveness of potential solutions. This approach can eventually decrease the high burden on healthcare-caused harm to the loss of capacity and productivity of patients being harmed, and to the loss of trust in the health system, and lead to additional available resources within the health system ^19^.

The impact of the project can be seen in different areas: research, education, clinical field, society, and policy.

The **research impact** is expressed in increased research excellence, improved scientific and innovation capabilities, and better performance by the ICM-UT and is revealed through the following indicators:

- increased number of peer reviewed publications in the field of PS and citations,
- increased visibility such as number of submitted, accepted and invited presentations in international events,
- new research topics and proposals which emerge from the project,
- successful participation in new national or EU level competitive research and innovation programs

The **educational impact** of the project is expressed new and improved courses with new educational methods available in person or online and integrated in the curricula of partner universities.

The expected long-term impacts of the project are seen as clinical, societal and policy impacts. The **clinical impact** is seen when PS practices are applied in clinical environment: patients will be treated by healthcare professionals who are trained in PS. Improvement of PS reporting and learning systems and implementation of evidence-based safety practices is expected to result in fewer adverse events and patient harm. The expected **societal impact** is expressed first of all in increased PS as a result of improvements following PATSAFE training. More information will be available on PS research, and this leads to deeper understanding of the role of patients in PS as well.

The project's **policy impact** is expressed in successful collaboration with stakeholders – providers of health services, the Estonian Health Insurance Fund, and the Ministry of Social Affairs, but also promoted by the national PS research strategy in Estonia. Moreover, this project provides an opportunity for researchers to partner with consumers to co-design patient safety research and healthcare services.

## Conclusion

As a result of the project, the ICM-UT will apply state-of-the-art evidence-based strategies to PS research. The ICM-UT has better capacity to conduct PS research using appropriate methodology, promote PS research among ESRs and healthcare staff, and involve patients in PS research, thus contributing to Estonia’s overall healthcare quality and PS performance. For partner institutions, participation in this project provides new opportunities for networking and expanding their research methods to a new culture and setting. Therefore, this innovative project will have the impact on the overall healthcare quality and safety. Bringing together national and international experts to exchange knowledge and experience will maximise the impact of the research for the benefit of the patients and clients of health system not only in Estonia but provides new knowledge and skill that could be implemented in other European countries as well.

## Data Availability

No data are associated with this article.
