# Supplementary material for: Developing the University of Tartu in Estonia into a well-networked Patient Safety Research Centre (PATSAFE): A study protocol
Source: Open Res Eur. 2023 Nov 24;2:107. Originally published 2022 Sep 5. [Version 2] doi: 10.12688/openreseurope.15024.2 (PMC11195608; doi:10.12688/openreseurope.15024.2)
Supplement: Peer review history

# Reviewer report: Reviewer response for version 2

**DOI:** 10.21956/openreseurope.18244.r41167

**Published:** 2024-07-11

**Authors:** Gianluca Catania

**Affiliations:** 1 Department of Health Sciences, University of Genoa, Genoa, Italy

Thank you for asking me to review the PATSAFE study protocol. The protocol aimed at improving and strengthening patient safety research initiatives addresses a relevant topic in healthcare systems. However there are some issues to be addressed. 

 Particularly the method section needs to be more expanded on how the objectives will be measured, also the authors should specify which outcome measures will be used for each outcome of the project.

 I would like to see a table with 3 columns:  left column the outcomes, central column the outcome measures, right column references for each measure (either original measure or adaptation of measure). It could be worth designing the table according to the four-level impact area defined by the authors (eg. research impact, educational impact,...).

 In the project concept section on page 6 it misses reference for the WHO guide for developing training programs in patient safety research.

 Data analyses section is missing.

 More than half of the references are more than 10 years old.

Is the study design appropriate for the research question?

Yes

Is the rationale for, and objectives of, the study clearly described?

Yes

Are sufficient details of the methods provided to allow replication by others?

No

Are the datasets clearly presented in a useable and accessible format?

No

Reviewer Expertise:

Nursing care organization

I confirm that I have read this submission and believe that I have an appropriate level of expertise to confirm that it is of an acceptable scientific standard, however I have significant reservations, as outlined above.

# Reviewer report: Reviewer response for version 2

**DOI:** 10.21956/openreseurope.18244.r41163

**Published:** 2024-06-29

**Authors:** Esther Van Poel

**Affiliations:** 1 Quality and Safety Ghent, Department of Public Health and Primary Care, Ghent University, Ghent, Belgium

Thank you for the opportunity to review this article. It presents the PATSAFE project within the Institute of Clinical Medicine of the University of Tartu (ICM-UT) as a leading institute for patient safety research and training in Estonia. A detailed SWOT analysis was conducted as a starting point for the project. A well-structured collaborative network is set up with international partners. Well-thought-out activities are mentioned. The project will have an impact on different fields, including research, education, clinical field, society, and policy. I agree with the project's importance, which could be inspiring for other countries.

 Suggestions for improvement

 1. " Estimates show that there are 421 million hospitalisations in the world annually, and approximately 42.7 million adverse events, i.e., one in 10 patients is harmed while receiving hospital care [...]." Are there any numbers on the European situation given the context of the Patient Safety Research Centre (PATSAFE) project? Please add more information on the consequences of unsafe care for different stakeholders (eg patients, healthcare providers,...)

 2. "The Faculty of Medicine of the University of Tartu is Estonia's only medical school. Within the Faculty, the Institute of Clinical Medicine (ICM-UT) is responsible for most of the clinical subjects of the Medicine programmes (except family medicine and dentistry) and has a leading role in clinical research [...]: Please add some references about your research etc.

 3."PS research is currently in an early stage, [...]": What do you mean?

 4. "We started the preparatory work for the Patient Safety Research Centre (PATSAFE) project by conducting a thorough SWOT analysis to identify the gaps in the scientific excellence in PS research methodology at ICM-UT.: " Who is 'we'?"

 5.Am I right that the project is solely focused on secondary care? Please make sure this is clear throughout the text and explain why.

 6. Please explain how you set up the collaboration with IQ Healthcare and UAB in Barcelona. Briefly introduce both institutions.

 7."In the short term, staff exchanges of two to three researchers from the ICM-UT will visit FAD and IQ-healthcare to identify and envisage research topics that are currently addressed at these institutions and the international level and how they are implemented. We identify the researchers who are interested in doing PS research or are already involved in PS research. Identification of research topics is based on the national and international priorities in PS research, by involving national and international level experts, i.e. representatives of researchers, practitioners, patients and policymakers. " Please add more information on the methodology you will use to identify international research priorities. How are you going to involve international experts etc.?"

 8. "3. Development of a joint research proposal and publications". Not only does the perception and readiness of clinicians seem important, but so does the care management team?

 9. "3. Development of a joint research proposal and publications." What about developing position statements with recommendations for policymakers etc?

 10. "As an exploitation strategy, the consortium takes advantage of the material, methodology, and experience developed during phases one, two, and three and organizes a virtual training course on PS research open to other potential interested professionals in Europe". Very useful initiative.

 11. "Conclusions": Who do you refer to by mentioning 'partnering institutions'?

 X.Please double-check typos and English grammar (e.g., one in ten patients are harmed...).

Is the study design appropriate for the research question?

Yes

Is the rationale for, and objectives of, the study clearly described?

Partly

Are sufficient details of the methods provided to allow replication by others?

Partly

Are the datasets clearly presented in a useable and accessible format?

Not applicable

Reviewer Expertise:

Patient safety - quality of healthcare

I confirm that I have read this submission and believe that I have an appropriate level of expertise to confirm that it is of an acceptable scientific standard, however I have significant reservations, as outlined above.

# Reviewer report: Reviewer response for version 2

**DOI:** 10.21956/openreseurope.18244.r41165

**Published:** 2024-06-18

**Authors:** Sofi Fristedt

**Affiliations:** 1 School of Health and Welfare, Jönköping University, Jönköping, Sweden

The protocol goes beyond a specific study to describe rather the development of a center. The introduction is relevant for the purpose of developing a center.

 The objectives are rather broad and cover a range of activities, and it will be difficult to assess whether they have been accomplished or not. They are more on a visionary level (enhancing, strengthening etc.) that will be hard to quantify or qualify. Bullet points in the SWOT analysis could perhaps be used as/to identify baselines - that will help in assessing fulfillment of the objectives.

 The design or rather the activities are rather overall described in relation to the activities. However, the protocol includes many activities and it is hard to describe them all in detail. The description is sufficient to see what they intend to do, but the description does not really support replication of the project. Maybe this format of a study protocol is not the best for such a large initiative as developing a center.

Is the study design appropriate for the research question?

Yes

Is the rationale for, and objectives of, the study clearly described?

Partly

Are sufficient details of the methods provided to allow replication by others?

Yes

Are the datasets clearly presented in a useable and accessible format?

Not applicable

Reviewer Expertise:

Former director of a research centre in quality improvement

I confirm that I have read this submission and believe that I have an appropriate level of expertise to confirm that it is of an acceptable scientific standard, however I have significant reservations, as outlined above.

# Reviewer report: Reviewer response for version 2

**DOI:** 10.21956/openreseurope.18244.r36420

**Published:** 2023-12-08

**Authors:** Anne Hogden

**Affiliations:** 1 University of New South Wales, Sydney, New South Wales, Australia

The revised version appears to address the issues identified in version 1. I am happy for version 2 to be accepted for indexing.

Is the study design appropriate for the research question?

Yes

Is the rationale for, and objectives of, the study clearly described?

Yes

Are sufficient details of the methods provided to allow replication by others?

Yes

Are the datasets clearly presented in a useable and accessible format?

Not applicable

Reviewer Expertise:

NA

I confirm that I have read this submission and believe that I have an appropriate level of expertise to confirm that it is of an acceptable scientific standard.

# Reviewer report: Reviewer response for version 1

**DOI:** 10.21956/openreseurope.16245.r32955

**Published:** 2023-07-10

**Authors:** Alexandra Peters

**Affiliations:** 1 University of Geneva, Geneva, Geneva, Switzerland

The authors address an important topic by looking to address the issue of patient safety in Estonia, especially in the context of the World Bank report which stated that Estonia needs to change its reporting culture to being open to acknowledging errors (p.4).

 I would recommend that the paper is revised before being Approved.

 Overall, the interventions remain vague and quite repetitive. I think the idea for identification of topics is good: staff exchanges, identification of topics, workshop for consensus of research topics and then a list of topics and methodologies. That said, the implementation of a training program is unclear. Is it just going to be advertised in the hospital generally where for “every course a flyer is developed and spread among potential participants : academic staff, ESRs, healthcare practitioners”. It seems that ICM-UT does not have the in house capacity to develop a course, and some staff exchanges is not going to give them the knowledge for course development. Will this be done with the other universities that they will collaborate with? All we know about the teachers is that they are “experts related to a topic in PS” (p9). There is no mention of the topics, types of experts that will teach, how to guarantee that people will attend, whether there is some kind of accreditation of mandatory participation to the courses, how long they will last, their scope, etc. Although the authors don’t have the topics yet, they could have gotten some of the broad topics for patient safety improvement form the literature.

 The three modules of the course on p9 mention three modules which include the fundamentals of PS, although on p6 it is specifically stated that the fundamental concepts of PS will not be focused on as they are discussed during the training of health professionals. (Although their course is not only targeting health professionals).

 The second half of the proposal has a number of grammatical errors (p.7 speaks about 2019-2022 being in the future (“will help ICM-UT address”), the last sentence on p8 is not a sentence, p9 “this team comprises of experts”, and “each module consists over several courses”.

 When speaking about improvement in the research number of papers and citations, it is very unclear how the 3 research proposals will be written, why there are 3, and what kind of proposals they are. There is no mention of implementing any types of interventions or anything. Although from what I understand, the main goal at this point is “to investigate the magnitude and nature of patient harm in Estonia and, ultimately promote the development of evidence-based strategies and evaluating the effectiveness of potential solutions” (sic), there is no concrete information about how their project will do this. I think that the authors should do a lot more research in patient safety in order to have a more solid project proposal. At the very least, they could identify very general categories such as reducing HAIs or reducing medical errors, etc and then begin to propose a more concrete project.

 Minor comments - although it is repeated that “knowledge of PS research methodology needs to be improved” in Estonia (p.4), there is often mention of “increasing visibility of ICM-UT’s excellence in PS research” (p4, p8, p9).

Is the study design appropriate for the research question?

Partly

Is the rationale for, and objectives of, the study clearly described?

Yes

Are sufficient details of the methods provided to allow replication by others?

No

Are the datasets clearly presented in a useable and accessible format?

Not applicable

Reviewer Expertise:

Infection prevention and control, healthcare environmental hygiene, hand hygiene

I confirm that I have read this submission and believe that I have an appropriate level of expertise to confirm that it is of an acceptable scientific standard, however I have significant reservations, as outlined above.

# Reviewer report: Reviewer response for version 1

**DOI:** 10.21956/openreseurope.16245.r32952

**Published:** 2023-07-10

**Authors:** Anne Hogden

**Affiliations:** 1 University of New South Wales, Sydney, New South Wales, Australia

The authors have provided a comprehensive protocol for the intended improvements to patient safety research. My comments are mostly about how this is communicated in the manuscript.

- The figures are excellent - very clear and to the point. They make the objectives very explicit, and link these well to the methodology.
- To improve the writing, some editing is required to reduce repetition and confusion within the text.
- The term 'soft skills' is used 7 times in the manuscript, but no definition is given. Please provide this explicitly on first use of the term.
- The acronym 'WP' is used in Figs 1 and 2, but not explained. Is it 'workshop'? There are several mentions of workshops in the text, but these need to be more overtly linked to the diagrams.
- Some questions - what will Estonia be able to teach the world through this project? What do you see as the implications for patients' safety and quality of care? You touch on this in the Long-term Impact section of Fig 3, but it would be good to see this taken a step further - how will patients benefit from this improved research? The benefits to researchers are clear, less so the benefits to patients.
- Participation in research is mentioned, but please consider this a wonderful opportunity for researchers to partner with consumers to co-design patient safety research and healthcare services.

Is the study design appropriate for the research question?

Yes

Is the rationale for, and objectives of, the study clearly described?

Yes

Are sufficient details of the methods provided to allow replication by others?

Yes

Are the datasets clearly presented in a useable and accessible format?

Not applicable

Reviewer Expertise:

Patient-centred care and co-design of research with patients and families; co-designed decision-making tools; lecturer in safety and quality in healthcare.

I confirm that I have read this submission and believe that I have an appropriate level of expertise to confirm that it is of an acceptable scientific standard, however I have significant reservations, as outlined above.
